# PepT1 mRNA expression levels in sea bream (*Sparus aurata*) fed different plant protein sources

**DOI:** 10.1186/2193-1801-2-17

**Published:** 2013-01-19

**Authors:** Genciana Terova, Lidia Robaina, Marisol Izquierdo, AnnaGiulia Cattaneo, Silvia Molinari, Giovanni Bernardini, Marco Saroglia

**Affiliations:** 1 Department of Biotechnology and Life Sciences, University of Insubria, Via J.H. Dunant, 3 - 21100, Varese, Italy; 2 Inter-University Centre for Research in Protein Biotechnologies “The Protein Factory”- Polytechnic University of Milan and University of Insubria, Varese, Italy; 3 Grupo de Investigación en Acuicultura, University of Las Palmas de Gran Canaria. Instituto Universitario de Sanidad Animal, Trasmontaña s/n, 35413 Arucas Las Palmas de Gran Canaria, Canary Islands

**Keywords:** Oligopeptide transporter PepT1, Gene expression, Real-time PCR, Fish meal substitution, Vegetable ingredients, Fish diet, Aquaculture

## Abstract

The expression and regulation of intestinal oligopeptide transporter (PepT)-1 when vegetable sources are used as a substitute for fish meal in the diet of marine fish has not yet been explored. In the present study, as part of our ongoing work on elucidating PepT1 gene expression in relation to different dietary treatments, we have now isolated and deposited in Genbank database (accession no. GU733710) a cDNA sequence representing the PepT1 in the sea bream (*Sparus aurata*). The “*de novo*” prediction of the three-dimensional structure of PepT1 protein is presented.

We also analyzed diet-induced changes in the expression of PepT1 mRNA via real-time RT-PCR using the standard curve method. Sea bream were fed for 140 days with one of the following four diet formulations (43% protein/21% lipid): a control fast growth-promoting diet (C), and three diets with the same formulation but in which 15% of the fish meal was substituted by protein concentrates either from lupine (LPC), chick pea (CPC), or green pea (PPC). Fish fed PPC had significantly (p < 0.05) lower levels of PepT1 transcripts in the proximal intestine than the controls, whereas PepT1 transcript levels in fish fed LPC or CPC were not significantly different from the controls. Although growth was similar between fish fed with different diets during the first 72 days of feeding, growth of the fish fed with PPC was reduced during the second part of the trial and was significantly (p < 0.05) lower than fish fed LPC and CPC diets by the end of the experiment. Correlation between these results and fish growth performances highlights that the intestinal PepT1 mRNA level may serve as a useful marker of the dietary protein quality and absorption efficiency.

## Introduction

The end products of intestinal protein digestion in both teleosts and mammals constitute a mixture of free amino acids and small peptides that are efficiently absorbed across the small intestinal epithelium (Clements & Raubenheimer 2006). Two distinct types of brush border membrane-associated transport systems are involved in this process: (1) amino acid transport, which may or may not be sodium dependent; and (2) transport of small peptides (2–3 amino acids) coupled to an H^+^ gradient. Intestinal peptide transport is of major nutritional significance in that the intraluminal products of protein digestion are predominantly di- and tripeptides, not amino acids as was widely believed over 30 years ago (Adibi 2003).

Cellular uptake of small peptides is mediated by H+/coupled peptide transporter (PepT1), located at the brush border membrane of intestinal epithelial cells (Yuen et al. 2007). PepT1 is a low-affinity, high-capacity transporter that mediates electrogenic uphill transport of di- and tripeptides from the apical membrane of the epithelial cells into the enterocytes. The transport is energized by a transmembrane electrochemical H + gradient directed from outside to inside. PepT1 has nutritional importance due to its dual role in intestinal absorption of a remarkable range of dietary protein-derived substrates and in reabsorption of peptide-bound amino nitrogen from glomerular filtrate in kidney (Daniel & Kottra 2004).

The gene encoding the proton-coupled di- and tripeptide transporter PepT1 was isolated first from rabbit small intestine. Fei et al. (Fei et al. 1994) cloned it in 1994 by microinjecting mRNA isolated from rabbit intestine into *Xenopus* oocytes, which resulted in functional expression of the protein. In subsequent years, PepT1 was characterized in great detail in higher vertebrates [mostly in mammals, but also in birds (for a review please see (Daniel et al. 2006). In contrast, information about this protein in lower vertebrates such as teleosts is limited, with the exception of PepT1 of the zebrafish (*Danio rerio*), Atlantic cod (*Gadus morhua*), Asian weatherloach, (*Misgurnus anguillicaudatus*), and sea bass (*Dicentrarchus labrax*). The functional activity and tissue expression pattern of PepT1 have been assessed in these species (Verri et al. 2003; Rønnestad et al. 2007; Gonçalves et al. 2007; Terova et al. 2009).

The affinity of PepT1 and its stereoselective capacity differ for different types of peptides, suggesting that its activity and expression may be modulated through the diet, particularly when different protein sources are utilized (Ostaszewska et al. 2010a; Ostaszewska et al. 2010b). However, to our knowledge, there is no information regarding the response at the mRNA level of such transporter when vegetable sources are used as a substitute for fish meal in the diet of marine fish.

Fish meal (FM) supply has become a limiting factor for the further development of fish feed production. Total annual world FM production is about 5–6 million tons (FAO 2010) and is predicted to remain stable for the next 10 years (Mazurkiewicz 2009). Increased FM cost, together with the limited availability, has encouraged the use of alternative sustainable sources of protein in fish feeds in the last few decades (Tacon & Metian 2008; Kaushik & Seiliez 2010). Thus, high rates of several terrestrial plant meals have been successfully included in the feed without affecting fish growth and production quality (Carter & Hauler 2000; Aslaksen et al. 2007; Sánchez-Lozano et al. 2011). Nevertheless, plant protein sources differ greatly in nutritional value, not only as regards their amino acid profiles and digestibility but also their carbohydrate content and characteristics or the processing method (Francis et al. 2001; Drew et al. 2007; Gatlin et al. 2007; Glencross et al. 2007). Therefore, the degree of success differs for different types of plant proteins. Among these sources, protein concentrates of pea (*Pisum sativum*), lupin (*Lupinus spp.*), or chick pea (*Cicer arietinum*) have been considered as good candidates for replacing fish meal. For instance, including up to 20% pea protein concentrate has been reported to support acceptable weight gain, feed intake, and feed conversion ratios in Atlantic salmon (Carter & Hauler 2000), rainbow trout (Thiessen et al. 2003), or sea bream (Sánchez-Lozano et al. 2011).

Accordingly, in the present study we first cloned a cDNA encoding PepT1 in gilthead sea bream and then assessed the impact of different feeds in which various vegetable sources substituted the fish meal on PepT1 mRNA levels in the proximal intestine of sea bream (*Sparus aurata*) with the aim to relate these expression levels to fish performance during the feeding trial.

## Materials and methods

### Feeds, fish and feeding protocol

Sea bream of about 92 g initial average weight were stocked into 500 litter fibreglass tanks at a density of 25 fish tank^-1^. Experiment was carried out in an open circulation system under natural photoperiod of about 12/12 dark/light. Temperature and oxygen concentrations measured during the trial ranged between 19.4 to 23.5°C and 6 to 7 ppm, respectively. Fish were fed to apparent satiation in triplicate groups for 140 days with one of the experimental diets. A fast growth promoting control diet (C) was prepared according to a basal commercial formulation (Dibaq Diproteg, Segovia, Spain) containing a regular composition of about 40% fishmeal and fish oil content and a mix of products and by products from cereals and oilseeds (50%), mainly soybean and wheat, and a remaining 10% for the vitamin and mineral premixes, antioxidants and antifungal components. A 15% of the fishmeal in this basal formula was substituted by protein concentrates from lupine (diet LPC), chick-pea (diet CPC) or green pea (diet PPC). All diets were produced at a commercial scale by Dibaq and were extruded throughout a 4 mm die size. Diets were isoproteic and isocaloric and their proximate composition is shown in Table 1. Feed biochemical composition analyses were conducted following standard procedures (AOAC. Available on line at: http://www.eoma.aoac.org/methods/search.asp?string=b). Dry matter content was determined after drying the sample in an oven at 105°C to constant weight, ash was determined by combustion in a muffle furnace at 600°C for 12 h, protein content (Nx6.25) was determined by Kjeldahl method and crude lipid was extracted following the method of Folch et al. (Folch et al. 1957).

**Table 1:** **Proximate composition of the experimental diets feed containing test ingredients**

	LPC	PPC	CPC
Protein (%)	46.02	45.37	45.30
Lipid (%)	20.83	20.64	20.04
Ash (%)	7.74	8.08	7.66
Moisture (%)	6.45	7.68	6.52

The fish were fed *ad libitum* 6 days a week in the following manner: food for each tank was weighed separately and placed in a container: the feed from each container was given in the morning (08:00) by hand, in small quantities until the fish ceased to respond; in the afternoon (15:00) the process was repeated; at the end of the day all the feed left in each container was weighed and the feed consumption of each tank was then registered. Feed conversion (g fish weight gain/g feed eaten) and specific feeding rates (daily fish feeding) were then calculated.

All fish were individually weighted at the beginning of the feeding trial and after 35, 72, 112 and 140 days of feeding the experimental diets. Weight data were analyses statistically by using the SPSS (13.1) statistical package. The level of statistical significance was set at 0.05. At the end of the trial, 5 fish from each tank were sacrificed. For the molecular biology analysis, the whole intestine was sampled and then dissected out in 7 equal segments of about 2 cm each. In addition, other tissues such as stomach, brain, muscle, and spleen were also isolated, frozen immediately in liquid nitrogen and then stored at the temperature of −80°C until the analysis. All procedures were approved by the Animal Care Committee of the University of Las Palmas de Gran Canaria and conducted according to the guidelines of the Italian Committee on Animal Care.

### Preparation of total RNA, cDNA synthesis, sea bream PepT1 cloning and sequencing

Total RNA was extracted from sea bream proximal intestine using PureYield RNA Midiprep System (Promega, Italy), following the protocol described in PureYield™ RNA Midiprep System Technical Manual #TM279, available online at: http://www.promega.com/tbs. This kit isolates intact, pure total RNA from essentially any sample type for use in a wide range of applications. The use of a novel Clearing Agent enables the rapid purification of total RNA with undetectable levels of genomic DNA contamination without using DNase. A novel combination of reagents, membranes and protocol leads to yields of up to 1 mg of total RNA without organic solvents, protease digestions or alcohol precipitations.

The quantity of the extracted RNA was calculated using the absorbance at 260 nm, whereas the integrity of RNA was assessed by agarose gel electrophoresis. Crisp 18S and 28S bands, detected by ethidium bromide staining were indicator of the intact RNA.

After extraction, total RNA was reverse transcribed into cDNA in a mix containing oligo dT16 primer, and dNTPs. This mix was heated, chilled on ice, and then reverse transcription buffer, DTT, RNaseOUT, and Moloney murine Leukaemia virus reverse transcriptase were added, as described in the M-MLV Reverse Transcriptase kit (Invitrogen).

To perform PCR, an aliquot of the resulting cDNA was amplified with GoTaq Polymerase (Promega) in a final volume containing buffer, dNTPs, and the primer set (sense + antisense) designed by us (Table 2). To design the primers, we firstly performed a BlastN search (http://www.ncbi.nml.nih.gov/BLAST/) for orthologues of PepT1 gene in other fish/vertebrate species. A multiple nucleotide sequence alignment (http://www.ebi.ac.uk/Tools/msa/clustalw2/) was then carried out on the sequences found and regions of strong nucleotide conservation were used to design the primers.

**Table 2:** **Sequences of the primers used in the work**

Primer	Sequence 5^′^– 3^′^	Purpose
PepT1-sense1	GATGACTTCGCCACCACTA	RT-PCR
PepT1-antisense1	CGATCAGATGCAGACGGTG	RT-PCR
PepT1-sense2	AGCAGGGCTCAAGATGGAC	RT-PCR
PepT1-antisense2	ACATCATCGTGCTCATCGTG	RT-PCR
PepT1_T7promoter	*gtaatacgactcactataggg*GGAGTGTGGTATTCACA	mRNA std.curve
PepT1_antisense3	AGCAGGGCTCAAGATGGAC	mRNA std.curve
PepT1 - sense	GCTACCCTCTGGCCTTTGG	Real-time
PepT1 - antisense	ATGGTGGTAGCTCTGATTGTGTTC	Real-time
Taqman PepT1	TCCCCGCTGCTCTC	Real-time

The PCR amplifications were performed for PepT1 sense1 + antisense1, and for PepT1 sense2 + antisense2 primer sets, using an automated Thermal Cycler (Mycycler, Biorad). An aliquot of each PCR reaction was then electrophoresed on agarose gel and bands were detected by ethidium bromide staining. The PCR products from PepT1 primer amplifications were then cloned using the pGEM®-T Easy cloning vector system (Promega, Italy) and subsequently sequenced in both directions (T7 and SP6).

### Quantitative real-time RT-PCR

#### Generation of in vitro-transcribed PepT1 mRNAs for standard curves

The number of PepT1 gene transcript copies were absolutely quantified by comparing them with a standard graph constructed using the known copy number of mRNA of this gene. For this, a forward (PepT1_T7promoter) and a reverse (PepT1_antisense3) primer were designed based on the mRNA sequences of *Sparus aurata* PepT1 after its identification (accession no. GU733710) (Table 2). This primer pair was used to create templates for the *in vitro* transcription of mRNAs for PepT1. *In vitro* transcription was performed using T7 RNA polymerase and other reagents supplied in the Promega RiboProbe *In Vitro* Transcription System kit according to the manufacturer’s protocol.

The molecular weight (MW) of the in vitro-transcribed RNA for each gene was calculated according to the following formula:

MW = (n° of A bases × 329.2) + (n° of U bases × 306.2) + (n° of C bases × 305.2) + (n° of G bases × 345.2) + 159.

The mRNAs produced by *in vitro* transcription were then used as quantitative standards in the analysis of experimental samples using one-step TaqMan EZ RT-PCR Core Reagents (Life technologies, Italy). RT- PCR conditions were: 2 min at 50°C, 30 min at 60°C, and 5 min at 95°C, followed by 40 cycles consisting of 20 s at 92°C, 1 min at 62°C. The Ct values obtained by amplification were used to create standard curves for target genes.

#### Quantitation of PepT1 transcripts by one-step RT-PCR TaqMan system

A hundred nanograms of total RNA extracted from the experimental samples was subjected, in parallel to 10-fold-diluted, defined amounts of standard mRNA, to real-time PCR under the same experimental conditions as for the establishment of the standard curves. Real-time Assays-by-Design^SM^ PCR primers and gene-specific fluorogenic probes were designed by Life technologies (LT). TaqMan® PCR was performed on a StepOne Real Time PCR System (LT, Italy). Data from Taqman® PCR runs were collected with StepOne’s Sequence Detector Program. The reaction efficiency was in the range 88–90%. Furthermore, a minus-reverse transcriptase control (“No Amplification Control” or NAC) was included in qRT-PCR experiments. The NAC was a mock reverse transcription containing all the RT-PCR reagents, except the reverse transcriptase. No product was seen in the NAC, which indicates that contaminating DNA was not present in the sample.

### Calculation and statistical analysis

The data were statistically compared using one-way analysis of variance (ANOVA). The level of statistical significance was set at p < 0.05.

### *In silico* analysis

The amino acid sequence of sea bream PepT1 (GenBank accession number GU733710.1) was analyzed using the open reading frame (ORF) finder program which is available at NCBI (http://www.ncbi.nlm.nih.gov). Nucleotide sequence was compared with other sequences available at the GenBank database using the BLAST algorithm (Altschul et al. 2003). Sequences were aligned using the ClustalW program (http://www.ebi.ac.uk/clustalw) and Multiple Sequence Alignments Editor & Shading Utility, GeneDoc, version 2.6.002 (http://www.psc.edu/biomed/genedoc).

#### Protein annotation

The partial putative amino acid sequence of sea bream (*Sparus aurata*) PepT1 (Genbank accession no. ADE58426.1) was compared with the complete sequence of sea bass (*Dicentrarchus labrax*) that we previously isolated (GenBank accession no. ACI49693.2) (Terova et al. 2009). Owing to the high similarity of the two sequences, the analyses for annotation at the MemPype server (http://mu2py.biocomp.unibo.it/mempype) (Pierleoni et al. 2011) and at the Interpro 33.0 (https://www.ebi.ac.uk/interpro/) (Hunter et al. 2012) were performed on the primary structure of *D. labrax* PepT1. We also submitted the structures to the SMART service for predicting similar domains (http://smart.embl-heidelberg.de/) (Letunic et al. 2009; Schultz et al. 1998).

#### Tertiary structure

The protein sequence of *sea bass* PepT1 aligns partially with only one structure present in the Protein Data Bank (PDB; entry: 2XUT; E-value: 8e-46) (http://www.pdb.org/pdb/home/home.do). As the alignment seems to be poor, especially after position 400 up to the C-terminus, its tertiary structure cannot be inferred from that of the template, and a “de novo” prediction of the tertiary structures of the putative proteins was obtained at the I-Tasser server (http://zhanglab.ccmb.med.umich.edu/I-TASSER). The four stages of the method implement a threading procedure followed by structural assembly, refinement of the model, and structure-based functional annotation. The output consists of five models, whose indexes of accuracy are the C score, the TM score, and the RMSD (Roy et al. 2010; Zhang 2007; Zhang 2008).

The tertiary structure-based COFACTOR algorithm predicted functional insights, including sites for ligand binding. We used the UCSF Chimera software, release 1.5.3 (http://www.cgl.ucsf.edu/chimera/) (Pettersen et al. 2004) to visualize, analyse, and compare the structural models.

#### Sites for post-translational modifications

A) Phosphorylation and sumoylation

The serine, threonine, and tyrosine phosphorylation sites were searched at the NetPhos 2.0 server (http://www.cbs.dtu.dk/services/NetPhos/) (Blom et al. 1999); sites for sumoylation were checked with the SUMOsp 2.0 program (http://sumosp.biocuckoo.org/) (Ren et al. 2009).

B) Glycosylation

The glycosylation sites possibly present in the proteins were searched at the server of the CBS (Center for Biological Sequence Analysis, at the Technical University of Denmark, (http://www.cbs.dtu.dk/services/). Sites for mucin-type mannosyl-O-glycosylation (Net-O-glyc 3.1) (Julenius et al. 2005), C-glycosylation (Johansen et al. 2006) and ε-glycosylation of lysine residues (Julenius 2007) were checked and searched. When sites for N-glycosylation were predicted at the CBS (Net-N-glyc 1.0) (Blom et al. 1999; Blom et al. 2004), a description of their tertiary structure was obtained with the GlyProt application at the Glycosciences server (http://www.glycosciences.de/modeling/).

## Results

### Sea bream PepT1 cDNA sequence

Sea bream PepT1 primer design was based on the alignment of four fish PepT1 coding sequences available on the NCBI Genbank database: *Dicentrarchus labrax* (accession no. FJ237043), *Gadus morhua*, (accession no. AY621934), *Sebastes nebulosus* (accession no. EU160494), and *Danio rerio* (accession no. AY300011). These presented several conserved regions within the sequence where primers could be reasonably designed.

**Figure 1:**
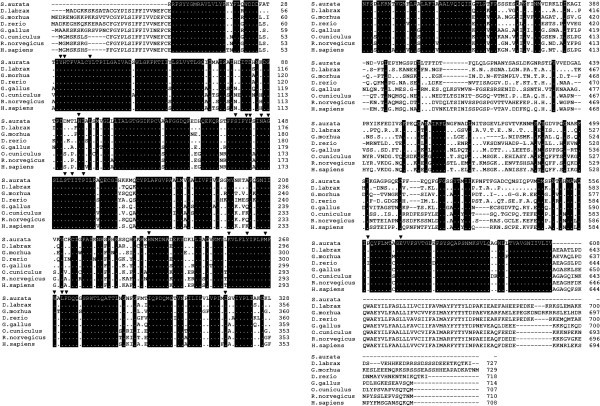
**Alignment of the deduced aminoacid sequence of sea bream (*Sparus aurata*) PepT1 (accession no.ADE58426) with the PepT1 related protein of sea bass (*Dicentrarchus labrax*) PepT1 (accession no.ACI49693) Atlantic cod (*Gadus morhua*) (accession no.AAY17354), zebrafish (*Danio rerio*) (accession no.AAQ65244), chicken (*Gallus gallus*) (accession no.NP_989696) rabbit (*Oryctolagus cuniculus*) (accession no.AAA21335), rat (*Ratus norvegicus*) (accession no.NP_476462), and human (*Homo sapiens*) (accession no.NP-005064).** Amino acids are designated by single-letter codes and are numbered to the right side. Dots indicate conserved residues in sea bass PepT1. Dashes indicate gaps introduced to facilitate alignment. Individual amino acid residues identified by site-directed mutagenesis in PepT1 proteins from various mammalian species and found to be relevant in determining the functional characteristics of the protein are indicated (black triangle) along the sequences.

Two cDNA fragments were obtained using PepT1 sense1+ antisense1 and PepT1 sense2 + antisense 2 (Table 2). Then, by connecting the sequences of the partially overlapping clones, a partial coding sequence of 1825 bp for sea bream PepT1 was determined. The cDNA for PepT1 was subsequently deposited in GeneBank under the accession no. GU733710. The deduced amino acid sequence shows that sea bream PepT1 is 608 amino acid long with a calculated molecular mass of approximately 81 kDa. Alignment of the amino acid sequence of sea bream PepT1 with that of other teleost, avian, mammalian, and primate species is shown in Figure 1. In the same figure are also indicated the positions of the highly conserved aminoacids.

### *In silico* analysis

The% similarities for alignments of PepT1 for different species, including the sizes of protein, are presented in Table 3. The amino acid sequence identity among the species was calculated using the open reading frame. Sea bream PepT1 showed the highest sequence similarity with teleost: (Sea bass: 88%; Yellow perch: 79%; Atlantic cod: 68%; Atlantic salmon, and zebrafish: 69%; spotted green pufferfish: 65%; China rockfish: 64%;) and avian species (chicken, and turkey: 64%; zebra finch: 60%), and lower homology with mammalian species (cattle: 62%; horse, common marmoset, rat, sheep, long tail macaque, and mouse: 61%; giant panda, dog, and pig: 60%; domestic rabbit: 58%).

**Table 3:** **Shared identities (%) between PepT1 proteins in different teleost, avian and mammalian species**

Gene	Species	GenBank accession number	Protein size (aa)	Identity with ***S. aurata*** (%)
	*Sparus aurata*	ADE58426	608	-
	*Dicentrachus labrax*	ACI49693	727	88
	*Perca flavescens*	ACX49753	729	79
	*Gadus morhua*	AAY17354	729	71
	*Salmo salar*	BAH24102	734	69
	*Danio rerio*	XP_001919914	717	69
	*Sebastes nebulosus*	ABV82968	742	67
	*Tetraodon nigroviridis*	CAG05928	671	65
**PepT1**	*Gallus gallus*	AAK39954	714	64
	*Meleagris gallopavo*	AAO16604	714	64
	*Taeniopygia guttata*	XP_002196515	790	60
	*Bos taurus*	DAA23771	707	62
	*Equus caballus*	XP_001493109	789	61
	*Callithrix jacchus*	XP_002742560	797	61
	*Ratus norvegicus*	BAA08844	710	61
	*Ovis aries*	AAK14788	707	61
	*Macaca fascicularis*	AAQ56235	708	61
	*Mus musculus*	AAI16249	709	61
	*Ailuropoda melanoleuca*	XP_002914915	707	60
	*Canis lupus familiaris*	AAL67837	708	60
	*Sus scrofa*	AAO43094	708	60
	*Oryctolagus cuniculus*	P36836	707	59

#### Protein annotation

Owing to the high similarity between the partial putative amino acid sequence of sea bream (*Sparus aurata*) PepT1 (Genbank accession no. ADE58426.1) and the complete sequence of sea bass (*Dicentrarchus labrax*) PepT1, the analyses for annotation at the MemPype server (http://mu2py.biocomp.unibo.it/mempype) (Pierleoni et al. 2011) and at the Interpro 33.0 (Hunter et al. 2009) were performed on the primary structure of sea bass PepT1. The complete range of bioinformatics analytical tools was used to characterize the PepT1 in this teleost.

**Figure 2:**
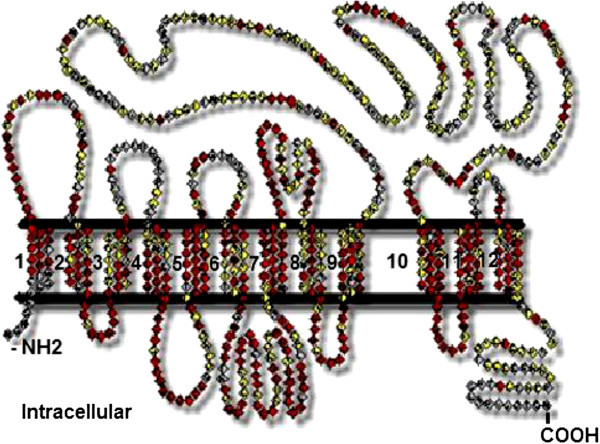
**Membrane spanning helices in sea bass PepT1 protein predicted with the THMM program (**http://www.cbs.dtu.dk/services/**).**

The 12 transmembrane domains of sea bass PepT1 protein, predicted by using the TMHMM program (http://www.cbs.dtu.dk/services/TMHMM/), are presented schematically in Figure 2. The Mempype server classified sea bass PepT1 as an internal membrane (confidence score = 95%). Similar entries were not found either in the SwissPro or Gene Ontology (GO) servers; therefore, we completed the search at the Interpro 3.3 server. The protein belongs to the proton/oligopeptide transporter 2 (PTR2) family. The corresponding Gene Ontology terms are: 0005215 (transporter activity); 0006857 (oligopeptide transport); and 0016020 (membrane). The PTR domain, recognized by PROSITE (http://prosite.expasy.org/), is located in the region between the 84^th^ and 627^th^ amino acids. It contains two subdomains: PTR2 (position: 73–97) and PTR3 (position 166–178).

#### Sites for post-translational modifications

**Figure 3:**
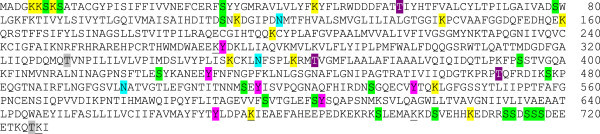
**Summary of all the predicted sites of post-translational modification found in PepT1 of*D.labrax*, shown on the primary sequence.** Colour code: phosphorylation indicated as: S= green, T= gray, Y= pink; glycosylation: N= blue, e= yellow and O= purple. Sumoylation sites are underlined.

Four sites of possible sumoylation are present at the following amino acid positions: 271, 676, 699, and 726. The PepT1 sequence contains several sites of phosphorylation along the entire chain (Ser: 68%, Thr: 11%, Tyr: 21%). No suitable sites for C-glycosylation were found. Thirteen sites of ε-glycosylation of lysine were predicted (positions 5, 6, 8, 45, 114, 143, 160, 199, 353, 362, 542, 676, and 707). The O-mannosylation was predicted at positions T330, and T725, and N-glycosylation at sites 120, 227, 357, 498, and 513. Five sites of N-glycosylation were predicted in the primary structure; however, only three of them (position 120, 227 and 357) seemed to be accessible in the tertiary structure of the molecule. It must be noted that, in the absence of the signal peptide, data concerning O- and N-glycosylation must be interpreted cautiously. All the features are highlighted along the primary structure in Figure 3.

#### Tertiary structure

**Figure 4:**
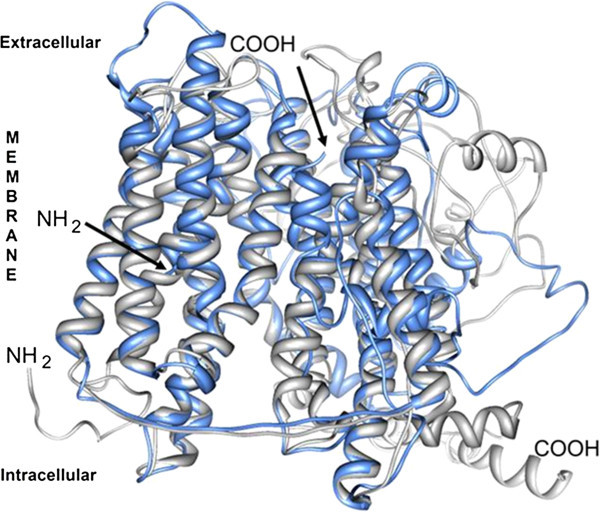
**The tertiary structures of the putative proteins PepT1 in*S.aurata*(partial, blue) and*D.labrax*(complete, gray) are aligned with the Chimera software.**

**Figure 5:**
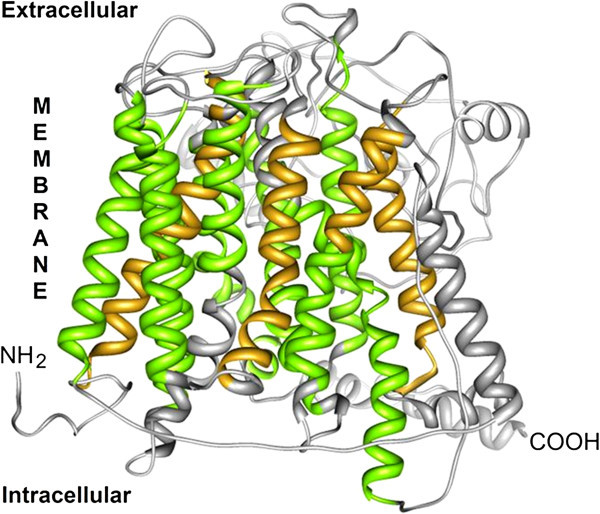
**The tertiary structure of the PepT1, predicted at the I-TASSER server, is characterized by 12 transmembrane domains, shaped as alpha helices, and longer enough to completely pass the all thickness of the lipid bilayer (60 vs 45 Å).** The protein starts and ends at the inner side of the bilayer. This feature is typical of channel proteins, to which the PepT1 belongs. A long chain not embedded in the bilayer is present. The transmembrane helices are here depicted in yellow or green, being the last one located inside the PTR2 domain.

**Figure 6:**
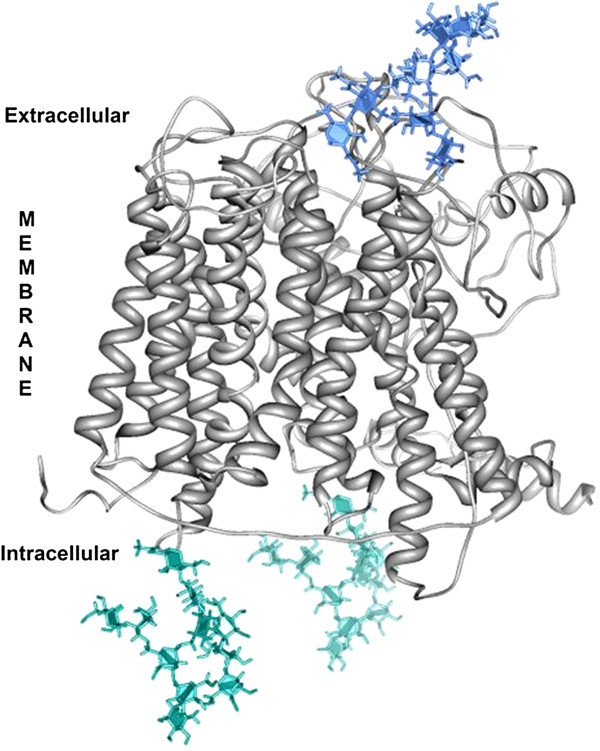
**A constructed 3D model of the PepT1 of*D.labrax,*in which the accessible N-glycosylation sites are bound to oligomannose.** Prediction obtained in silico by the GlyPro (http://www.glycosciences.de/modeling/).

**Figure 7:**
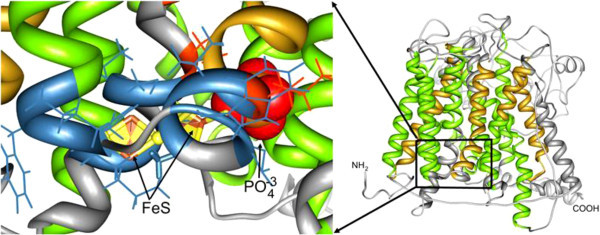
**The sites of binding for organic acids and metals in the PepT1 of*D.labrax*, as predicted by the COFACTOR algorithm.** The 3D structure inside the box on the right of the figure was zoomed and rotated to be viewed from the citoplasmic side of the protein, focusing on the binding regions. Blue: residues binding metal clusters, red: residues binding phosphate.

The second model obtained at the I-TASSER server was more consistent with the spatial arrangement of the transmembrane regions and the intra- and extracytoplasmic loops of PepT1 in both fish species taken into consideration. Sea bass and sea bream structural alignment (Figure 4) is largely acceptable; therefore, we used the 3D structure of PepT1 protein obtained in *D. labrax* as representative for both species. Sea bass Pept1, like all members of the proton-coupled oligopeptide transporter superfamily, is predicted to traverse the membrane 12 times, with amino and carboxyl termini facing the cytosol, and with an elongated extracellular loop connecting the ninth and tenth transmembrane segments (Figures 4, and 5). Several binding sites such as those for phosphate ions and two different types of iron clusters (Figure 6) were predicted based on the similarities with the binding sites of the crystallographically resolved PepT_So_ protein from *Shewanella oneidensis* (Newstead et al. 2011). In Figure 7 are shown three accessible sites for N-glycosylation, reconstructed “*in silico*” with the addition of oligomannose.

### Fish growth and feeding rates

**Figure 8:**
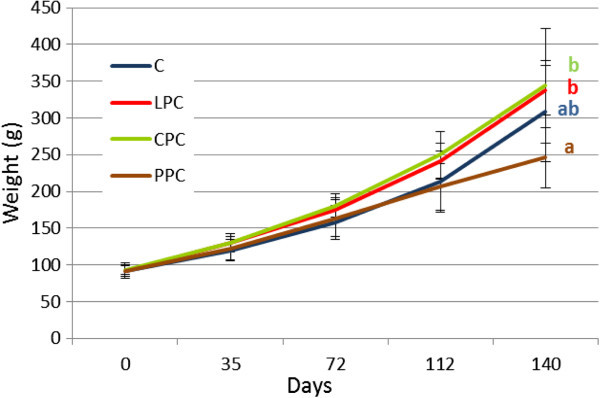
**Sea bream body weight during the experiment.** Fish were fed for 140 days with one of the following four diet formulations (43% protein/21% lipid): control fast growth-promoting diet (C) and three diets with the same formulation but in which 15% of the fish meal was substituted by protein concentrates either from lupine (LPC), chick pea (CPC), or green pea (PPC). Significant differences (p < 0.05) were only observed for fish body weight after 140 days of feeding.

Including plant protein concentrates did not affect feed acceptance. Therefore, we did not find significant differences in total feed intake between diets, with specific feeding rates ranging from 1.06 to 1.18 for the entire period. Although growth was similar between fish fed with different diets during the first 72 days of feeding (Figure 8), growth of the fish fed with PPC was reduced during the second part of the trial and was significantly lower (p < 0.05) than fish fed LPC and CPC diets only at the end of the experiment (Table 4). Subsequently, average feed conversion index was significantly poorest for diet PPC (2.25), in comparison to the other 3 diets (1.90 for LPC and 1.91 for CPC, and C).

**Table 4:** **Fish weight (g) at the end of the trial after feeding the experimental diets**

	C	LPC	CPC	PPC
Fish weight (g)	309.25 ± 31.90^ab^	338.30 ± 43.48^b^	343.67 ± 37.87^a^	245.77 ± 37.68^a^

Different letters denote statistically significant differences (p < 0.05) between treatments.

### Spatial distribution of PepT1 mRNAs in sea bream digestive tract

Total RNA from sea bream tissues was subjected to real-time RT-PCR using the standard curve established for PepT1 mRNA to determine absolute amounts of PepT1 mRNA. To create the standard curve, the correct template length including the T7 promoter was verified by 2% agarose gel electrophoresis. Quality and purity of mRNAs were confirmed by the ratio of absorptions at 280/260 nm, i.e., 1.8-2.0 (data not shown). To obtain threshold cycle (Ct) values for the target gene, defined quantities at 10-fold dilutions of PepT1 mRNAs were subjected to a one-tube two-time real-time RT-PCR. The standard curves created for PepT1 were based on the linear relationship between the Ct value and the logarithm of the starting amount.

**Figure 9:**
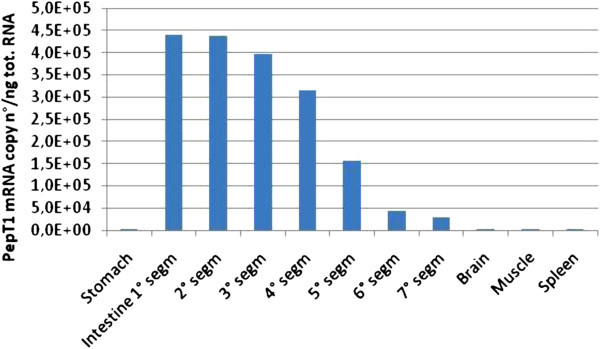
**Spatial distribution of sea bream PepT1 mRNA in the digestive tract as determined by Real-Time quantitative PCR.** PepT1 mRNA copy number was normalized as a ratio to 100 ng total RNA.

This analysis revealed the following spatial distribution of PepT1 gene expression in sea bream digestive tract: high levels of expression in segments 1–3 (the first 3 cm) of the proximal intestine, lower levels in intestinal segments 4–7, and very low levels in stomach, brain, spleen and muscle (Figure 9)

### PepT1 mRNA copy number in sea bream proximal intestine during the experiment

**Figure 10:**
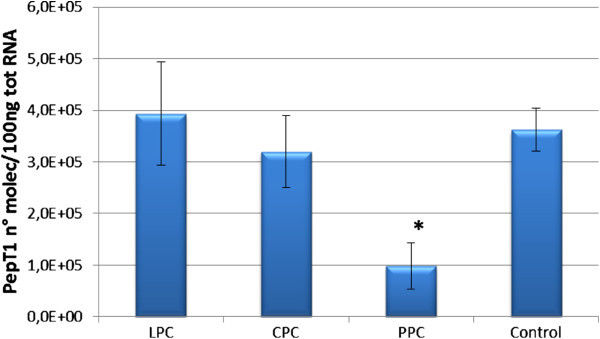
**Expression levels of PepT1 measured by real-time PCR in*S. aurata*proximal intestine during the experiment.** PepT1 mRNA copy number was normalized as a ratio to 100 ng total RNA. Bars indicate standard error of the mean. Differences were determined by one-way analysis of variance (ANOVA). (*) indicates significantly different means (*p* < 0.05).

The absolute mRNA levels of PepT1 in the proximal intestine of sea bream in response to the feeding trial are presented in Figure 10. As shown, fish fed for 140 days with a diet comprised of 43% protein and 21% lipid in which 15% of the fish meal was substituted by protein concentrates from green pea (PPC) had significantly lower levels of PepT1 transcripts (p < 0.05) in the proximal intestine than the controls. PepT1 transcript levels in fish fed the same diet (43% protein / 21% lipid) with 15% of the fish meal substituted by protein concentrates from lupine (LPC) or chick pea (CPC) were not significantly different from the controls.

## Discussion

A cDNA clone representing the PepT1 in sea bream (*Sparus aurata*) was successfully identified in the present study. The predicted sea bream PepT1 amino acid sequence shows extensive sequence similarity to human and other vertebrate PepT1.

Several functionally important sequence motifs described in all known vertebrate PepT1 proteins are also found in the sea bass (Figure 1). They include:

1. The highly conserved histidine residue (H57) (numbering according to human PepT1) (Terada et al. 1996; Chen et al. 2000; Fei et al. 1997). This extracellular residue is considered to be of central importance for PepT1 activity. Indeed, PepT1 was shown to be efficiently inactivated by diethylpyrocarbonate as a histidine-modifying agent (Terada et al. 1996), and mutation of H57 completely inactivated the transporter (Fei et al. 1997; Uchiyama et al. 2003).
2. The H57 adjacent tyrosine residue (Y56), which was also shown to be involved in substrate interaction by reducing the affinity for differently charged dipeptides when mutated to a phenylalanine (Chen et al. 2000).
3. A tyrosine residue (Y64), which appears to be involved in substrate translocation since the subtle change to a phenylalanine residue almost, abolished transport (Chen et al. 2000).
4. Tyr-167, part of the PTR family signature motif, which also seems to be essential for PepT1 activity (Yeung et al. 1998). It is thought to have an essential role in dipeptide uptake (due to the unique chemistry of its phenolic side chain). In fact, replacing it with Ala, Phe, His, Ser, and Cys abolished Gly-Sar uptake of PepT1 (Yeung et al. 1998; Bolger et al. 1998; Kulkarni et al. 2003a).
5. His-121 and His-260, which have been studied together with H111 (H111 is replaced with Lys in sea bream, sea bass, cod, zebrafish, and chicken) due to the pH dependence of the transport processes. However, mutations did not yield convincing evidence for an essential role in the transport process (Chen et al. 2000; Fei et al. 1997).
6. The highly conserved Ser-164, L168, G173, and I179. These amino acids are responsible for incorrect packaging and/or transport of the protein to the plasma membrane. In fact, the respective mutants S164C, L168C, G173C, and I179C were not expressed on the HEK293 plasma membrane (Kulkarni et al. 2003a).
7. Asn 171, Ser 174, and P182, which play a critical role in substrate binding. The mutations N171C and S174C, respectively, abolished Gly-Sar uptake of PepT1, whereas the mutant P182C showed only 40% of uptake (Kulkarni et al. 2003a).
8. Arg-282, which in sea bream and other teleosts is replaced with another positively charged amino acid (Lys). It seems that the positive charge is important at this amino acid position. A salt bridge between R282 and D341 may play a role in maximizing the efficiency of substrate translocation. Mutations R282A, R282C, and R282K have a modest effect on Gly-Sar uptake, whereas mutations R282E and R282D show significantly reduced uptake of Gly-Sar (Bolger et al. 1998; Kulkarni et al. 2003b; Kulkarni et al. 2007).
9. Tyr-287 and Met-292, which play critical roles in synthesis and/or folding of the protein. Single cysteine mutation at these positions (Y287C and M292C) was responsible for misfolding of the mutated protein (Kulkarni et al. 2003b).
10. Trp-294, which plays a role in maintaining the structural integrity of the protein. The mutant W294A showed reduced uptake (~ 8%) of Gly-Sar and mutation had a significant effect on the Michaelis-Menten *K*m value, whereas W294C did not show any reduction in Gly-Sar uptake (Bolger et al. 1998; Kulkarni et al. 2003b). The larger size of the cysteine side chain, compared with that of alanine, sufficiently reflects the steric bulk of the tryptophan side chain and better maintains the correct helical packing.
11. Lys-296 and Phe-297. These residues probably play a structural role in transporter function, as mutants L296C and F297C display negligible uptake of Gly-Sar (Kulkarni et al. 2003b).
12. The highly conserved Asp-341. It seems that the negative charge is important at this amino acid position. A salt bridge between R282 and D341 may play a role in maximizing the efficiency of substrate translocation. Mutations D341A and D341E have a modest effect on Gly-Sar uptake, whereas mutations D341K and D341R show significantly reduced uptake of Gly-Sar (Kulkarni et al. 2007).
13. Pro-586, which may have profound effects on translation, degradation, and/or membrane insertion. Mutant P586L showed reduced transport capacity, lower protein levels, and lower plasma membrane expression (Zhang et al. 2004).
14. Glu-595. Mutant E595A showed reduced uptake of Gly-Sar (Bolger et al. 1998).

The overall high degree of PepT1 sequence conservation through evolution is not only consistent with its essential role for growth and metabolism, but also suggests that its biological action may be equally well conserved. In fact, numerous aspects of digestion and absorption in fish and mammals are similar, demonstrating high conservation of these mechanisms during evolution.

The partial coding sequence of *S. aurata* PepT1 seems to be very similar to the PepT1 of *D. labrax* and the two structures align well. The latter is an integral membrane protein, characterized by a series of 12 transmembrane helices, and by a long loop at the external side of the membrane. Both the N- and C- terminal are in the cytoplasmic environment. The number of predicted transmembrane regions is 12 instead of 14 of the PepTSo obtained in a prokaryote (Newstead et al. 2011); otherwise, sea bass PepT1 aligns well, although partially, with the template of PepTSo in the PDB repository (2XUT, structural alignment not shown), The length of the helices is approximately 60 Å, a value consistent with the lipid bilayer thickness (45–50 Å). Other features of the two structures also seem to be quite similar, such as the long extramembrane loop at the outer side of the lipid bilayer connecting the ninth and tenth transmembrane segments which is positioned between aa 386 and 581 in PepT1 of *D. labrax*.

In addition to the transmembrane helices, which characterize the structure as a channel, a PTR2 domain confers on it the features of proton/oligopeptides symporters. The PTR2 subdomain (position 166–178) has been characterized in the crystallographically resolved structure of the PepTSo of *S.oneidensis*, and several binding sites predicted in the structure of *D.labrax* correspond with those found by the authors who conducted the crystallographic work (Newstead et al. 2011). In this domain fall all the predicted binding sites for phosphate ions and iron clusters.

Sites for post-translational modification have been predicted along the PepT1 protein, including sites of sumoylation, phosphorylation, and ε-, O-, and N-glycosylation. The two latter ones should be considered with caution, however, because a signal peptide is lacking. Indeed, only one of the three sites, the one at position 120, could be linked to sugars, this being the only region extending to the extracellular environment, the site of glycosylation in eukaryotes.

In the present work we used real-time PCR technology to carefully assess the impact of dietary manipulation on PepT1 mRNA expression levels in the intestine of sea bream, a marine fish with desirable features for aquaculture and one that is widely cultivated in the Mediterranean area. Our results demonstrate that PepT1 is abundantly expressed in the proximal intestine and pyloric ceca of this species, but it is virtually absent or expressed at very low levels in the stomach and in the distal part of intestine. Thus, it is also likely that, in sea bream, the proximal intestine represents the main expression and production site for PepT1, as also demonstrated in humans (Ford et al. 2003) and other fish species such as sea bass (Terova et al. 2009), cod (Rønnestad et al. 2007), weatherloach (Gonçalves et al. 2007), and zebrafish (Verri et al. 2003). The presence of PepT1 mRNA in the subsequent 10-cm region of the intestinal tissue, at albeit differing levels, is similar to the spatial distribution of PepT1 mRNAs found in sea bass (Terova et al. 2009) and Atlantic cod intestine (Rønnestad et al. 2007). In addition, the relatively very low level of expression in sea bream stomach matches the pattern reported for sea bass and Atlantic cod.

To study the dietary regulation of PepT1 mRNA levels in *S. aurata,* fish were fed protein from four different sources. After 140 days of feeding, fish fed a diet containing 15% green pea meal showed a downregulation of PepT1 mRNA levels, together with a lower growth than fish fed the same percentage of either lupin or chick pea meals in the diet. Although field peas (*Pisum sativum*) have been found to be a promising protein source for several species, such as Atlantic salmon (Aslaksen et al. 2007; Øverland et al. 2009), rainbow trout (Thiessen et al. 2003), sea bass (Gouveia & Davies 1998), and sea bream (Sánchez-Lozano et al. 2011), its effectiveness must be related to fish size and species, dietary inclusion levels, dietary fish meal content, or raw material processing conditions. For instance, including 10% untreated pea meal or 20% pea protein isolate in diets for juvenile milkfish (*C. chanos*) (Borlongan et al. 2003) or tilapia (*O. niloticus*) (Schulz et al. 2007) negatively affected fish performance, whereas up to 40% whole pea meal (Gouveia & Davies 1998) or up to 30% extruded pea meal (Gouveia & Davies 2000) has been successfully included in sea bass diets and up to 37% extruded pea meal (Pereira & Oliva-Teles 2002) or 32% pea protein concentrate in extruded diets (Sánchez-Lozano et al. 2011) in gilthead sea bream. In the present study, 15% pea protein concentrate tended to reduce growth performance of sea bream, whereas in previous studies conducted in the same species (Sánchez-Lozano et al. 2011), with the same pea protein concentrate and under similar fish size and temperature conditions, it did not affect growth. These different results could be related to the different protein and amino acid profiles of the two diet formulations since, for instance, the latter study included higher amounts of fishmeal.

Interestingly, higher amounts of dietary pea protein concentrate reduce both digestible protein and essential amino acid retention in sea bream (Sánchez-Lozano et al. 2011), which might be at least partially related to a lower di-and tri-peptide transport in the anterior intestine as suggested by the downregulation of PepT1 gene expression in the present study. Furthermore, indigestible carbohydrates, mainly nonstarch polysaccharides (NSPs), are part of the cell wall structure of legumes, reducing feed utilization and growth performance in fish. NSPs are composed predominantly of linked monomers of hexoses and pentoses, such as galactose, glucose, or xylose (Van Barneveld 1999), whereas enzymes such as β-glucanases or β-xylanases that digest NSPs are scarce or nonexistent in fish (Kuz’mina 1996). Consequently, the dietary NSPs remain indigestible in fish and may reduce the gastrointestinal passage rate of the food (Storebakken et al. 1999) and interfere with digestion of other dietary nutrients (Storebakken & Austreng 1987; Refstie et al. 1999). Only very recently, it was found that high inclusion levels of pea protein concentrate (35%) negatively affect gut health in Atlantic salmon, reducing brush border enzyme activities in the distal intestine of the fish and causing diarrhea associated with a reduction in mucosal fold height and increase in *lamina propria* width and infiltration (Sinha et al. 2011). Moreover, the presence of saponins in pea protein concentrate, which is highly resistant to the ingredient condition processing (Sinha et al. 2011), also affects gut integrity (Knudsen et al. 2008). Therefore, altered gut integrity caused by several antinutrients found in pea protein concentrate could also be related to the lower expression of PepT1 found in sea bream in the present trial. Further studies are being conducted to clarify this hypothesis.

In conclusion, a cDNA sequence encoding PepT1 in sea bream (*Sparus aurata*) was isolated and characterized in the present study. The study also demonstrated that PepT1 mRNA copy number is modulated by dietary protein sources: including 15% green pea protein concentrate “downregulates” the levels of PepT1 gene expression in comparison to 15% lupine or chick pea protein concentrate.
